# Non-invasive Vagus Nerve Stimulation for COVID-19: Results From a Randomized Controlled Trial (SAVIOR I)

**DOI:** 10.3389/fneur.2022.820864

**Published:** 2022-04-08

**Authors:** Carlos Tornero, Ernesto Pastor, María del Mar Garzando, Jorge Orduña, Maria J. Forner, Irene Bocigas, David L. Cedeño, Ricardo Vallejo, Candace K. McClure, Christopher J. Czura, Eric J. Liebler, Peter Staats

**Affiliations:** ^1^Hospital Clínico Universitario de Valencia, Anesthesia, Critical Care and Pain Management Unit, Valencia, Spain; ^2^Cátedra Dolor, UFV-Fundación Vithas, Madrid, Spain; ^3^Hospital Clínico Universitario de Valencia, Internal Medicine Department, Valencia, Spain; ^4^Hospital Clínico Universitario de Valencia, Pulmonary Department, Valencia, Spain; ^5^Department of Basic Science, Millennium Pain Center, Bloomington, IL, United States; ^6^Department of Psychology, Illinois Wesleyan University, Bloomington, IL, United States; ^7^NAMSA, Minneapolis, MN, United States; ^8^Convergent Medical Technologies, Inc., Oyster Bay, NY, United States; ^9^electroCore, Inc., Rockaway, NJ, United States

**Keywords:** COVID-19, coronavirus, non-invasive vagus nerve stimulation, neuromodulation, respiratory symptoms, randomized control trial, biomarkers, inflammation

## Abstract

**Background:**

Severe coronavirus disease 2019 (COVID-19) is characterized, in part, by an excessive inflammatory response. Evidence from animal and human studies suggests that vagus nerve stimulation can lead to reduced levels of various biomarkers of inflammation. We conducted a prospective randomized controlled study (SAVIOR-I) to assess the feasibility, efficacy, and safety of non-invasive vagus nerve stimulation (nVNS) for the treatment of respiratory symptoms and inflammatory markers among patients who were hospitalized for COVID-19 (ClinicalTrials.gov identifier: NCT04368156).

**Methods:**

Participants were randomly assigned in a 1:1 allocation to receive either the standard of care (SoC) alone or nVNS therapy plus the SoC. The nVNS group received 2 consecutive 2-min doses of nVNS 3 times daily as prophylaxis. Efficacy and safety were evaluated *via* the incidence of specific clinical events, inflammatory biomarker levels, and the occurrence of adverse events.

**Results:**

Of the 110 participants who were enrolled and randomly assigned, 97 (nVNS, *n* = 47; SoC, *n* = 50) had sufficient available data and comprised the evaluable population. C-reactive protein (CRP) levels decreased from baseline to a significantly greater degree in the nVNS group than in the SoC group at day 5 and overall (i.e., all postbaseline data points collected through day 5, combined). Procalcitonin level also showed significantly greater decreases from baseline to day 5 in the nVNS group than in the SoC group. D-dimer levels were decreased from baseline for the nVNS group and increased from baseline for the SoC group at day 5 and overall, although the difference between the treatment groups did not reach statistical significance. No significant treatment differences were seen for clinical respiratory outcomes or any of the other biochemical markers evaluated. No serious nVNS-related adverse events occurred during the study.

**Conclusions:**

nVNS therapy led to significant reductions in levels of inflammatory markers, specifically CRP and procalcitonin. Because nVNS has multiple mechanisms of action that may be relevant to COVID-19, additional research into its potential use earlier in the course of COVID-19 and its potential to mitigate some of the symptoms associated with post-acute sequelae of COVID-19 is warranted.

## Introduction

The clinical presentation of coronavirus disease 2019 (COVID-19) is highly variable, ranging from asymptomatic to critical multisystem failure (1). Initial symptoms of COVID-19 commonly include fever and cough that may progress to respiratory distress; supportive treatment with antipyretics, antitussives, and supplemental oxygen is often the standard of care (SoC) for these symptoms (1–3). Adults and children with mild to moderate symptoms of COVID-19 who are at risk for progressing to severe COVID-19 and/or hospitalization may receive treatment with neutralizing antibody drugs like bamlanivimab and etesevimab under an emergency use authorization (EUA) (4). Severe cases of respiratory distress may require hospitalization and non-invasive and/or invasive mechanical ventilation (3). Adults and children who are hospitalized and require supplemental oxygen, mechanical ventilation, or extracorporeal membrane oxygenation may also be given the glucocorticoid dexamethasone in combination with baricitinib and remdesivir (5, 6).

As the treatment of COVID-19 evolved to include the use of glucocorticoids, it became apparent that the pathologic sequelae of COVID-19 are due, in part, to an acute, excessive inflammatory response. The inflammatory reflex, a key function of the vagus nerve, modulates the host response to bacterial and viral infections (7, 8). In animal models of sepsis, electrical vagus nerve stimulation (VNS) has been shown to decrease the release of pro-inflammatory cytokines, attenuate multiple organ dysfunction, and improve survival (9–11). In humans, VNS has been demonstrated to reduce or prevent increases in circulating levels of proinflammatory cytokines, including tumor necrosis factor (TNF)-α, interleukin (IL)-1β, IL-6, and interferon-γ, with the specific cytokines affected varying among the disease states studied (12–15). These observations support the hypothesis that inhibition of inflammation with VNS could play a role in the clinical care of patients hospitalized for COVID-19. Owing to the invasive nature of vagus nerve stimulator implantation, historically, the applicability of VNS in critical care medicine was limited.

Non-invasive vagus nerve stimulation (nVNS) in this study was delivered by a handheld, portable vagus nerve stimulator (gammaCore Sapphire™, electroCore, Inc.) that delivers transcutaneous electrical stimulation to cervical vagus nerve fibers and is US Food and Drug Administration (FDA) approved for the prevention and acute treatment of migraine and cluster headache and Conformité Européenne (CE) marked in the EU for the prevention and treatment of symptoms of reactive airway disease and other conditions. Studies of nVNS in posttraumatic stress disorder and Parkinson disease have suggested that it can reduce systemic levels of inflammatory biomarkers in these conditions (14, 15).

We hypothesized that the administration of nVNS in the hospital setting could inhibit the inflammatory signaling process indicative of COVID-19 progression and potentially improve breathing. Here we report feasibility, efficacy, and safety findings from an initial prospective randomized controlled trial of nVNS for the treatment of respiratory symptoms of COVID-19 and inflammatory markers in hospitalized patients (ClinicalTrials.gov identifier: NCT04368156).

## Methods

### Participants

This prospective randomized controlled study was conducted at Hospital Clínico Universitario de Valencia (Valencia, Spain). The clinical research plan was reviewed and approved by an Institutional Review Board and an Ethics Committee before patient enrollment. Research was conducted in accordance with the Declaration of Helsinki. All patients provided written informed consent before participation.

Investigators enrolled patients ≥18 years of age who were admitted to the hospital, had positive test results for COVID-19 infection (via severe acute respiratory syndrome coronavirus 2 polymerase chain reaction testing), and were experiencing cough and respiratory involvement. Inclusion and exclusion criteria are further described in Table 1. After randomization, demographics and medical histories of participants were compiled and analyzed.

**Table 1:** Inclusion and exclusion criteria.

**Inclusion criteria**
1. ≥18 years old 2. Positive results for COVID-19 with cough and respiratory involvement 3. Oxygen saturation ≥92% without the need for mechanical ventilation or severe respiratory insufficiency that will require immediate intubation 4. Agrees to use the nVNS device according to the instructions and will follow the requirements of the study 5. Able to provide written informed consent
**Exclusion criteria**
1. Pregnant 2. Requires home oxygen therapy at the start of the study and before the development of COVID-19 3. Already enrolled in a clinical trial for COVID-19 4. History of aneurysm, intracranial hemorrhage, brain tumor, or significant head trauma 5. Known or suspected severe atherosclerotic cardiovascular disease, severe carotid artery disease, congestive heart failure, known severe coronary artery disease, or recent myocardial infarction 6. Uncontrolled high blood pressure 7. Presence of an implanted electrical and/or neurostimulator device, metal cervical spine material, or a metal implant near the site of nVNS 8. Is from a vulnerable population or has a condition that affects their ability to provide informed consent or comply with follow-up requirements or has compromised their ability to provide self-assessment

*COVID-19, coronavirus disease 2019; nVNS, non-invasive vagus nerve stimulation*.

### Randomization

**Figure 1:**
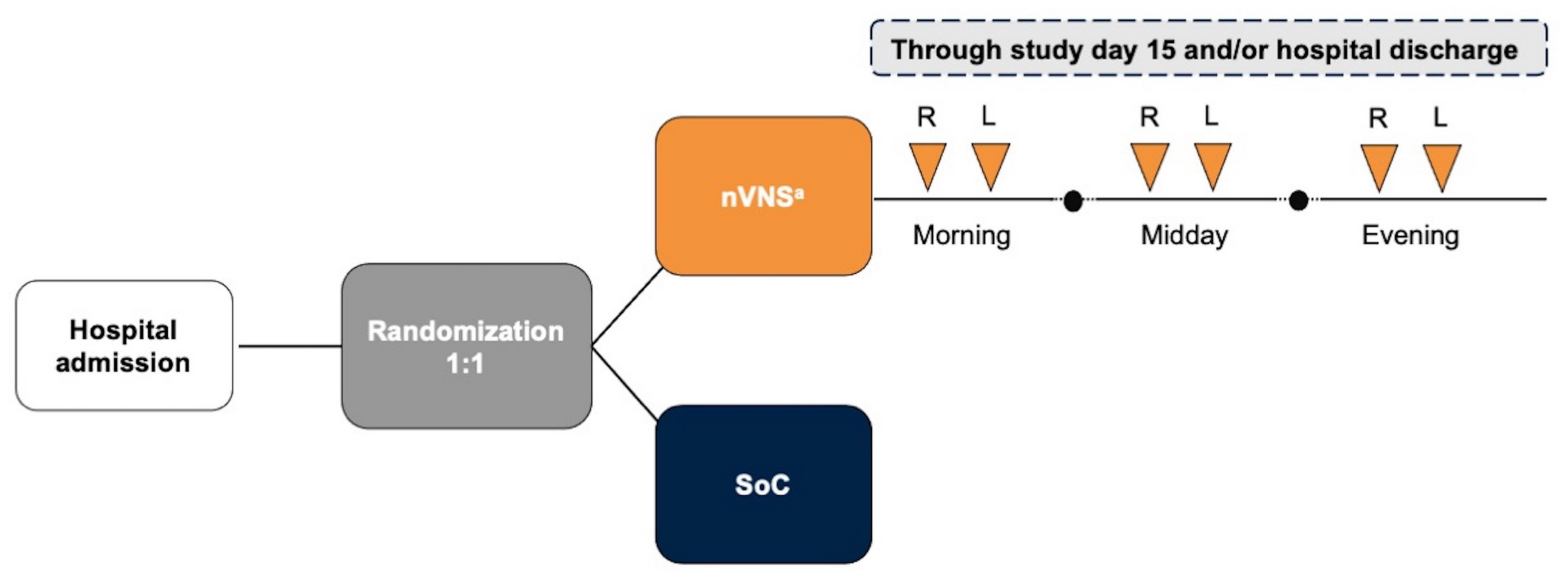
Study Design. ^a^Each stimulation was 2 min in duration. L, left side; nVNS, non-invasive vagus nerve stimulation; R, right side; SoC, standard of care.

After providing informed consent, participants were randomly assigned in a 1:1 allocation to receive either the routine SoC alone or treatment with nVNS in addition to the SoC (Figure 1).

### Procedures

**Figure 2:**
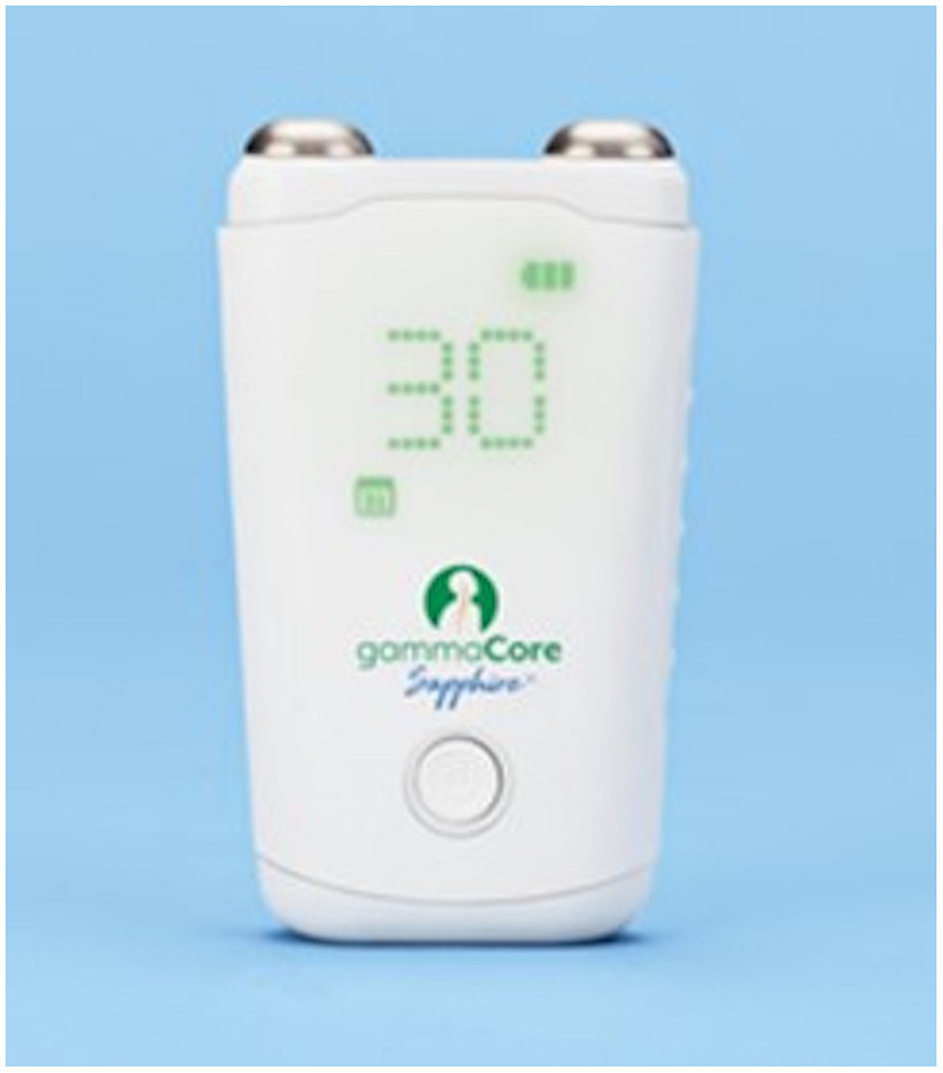
The nVNS Device (gammaCore Sapphire™). Image provided courtesy of electroCore, Inc. nVNS indicates non-invasive vagus nerve stimulation.

In the active treatment group, nVNS was administered using gammaCore Sapphire™ (electroCore, Inc.; Figure 2), which delivers a low-voltage electrical signal consisting of a 5-kHz sine wave burst lasting for 1 ms (5 sine waves, each lasting 200 ms), with such bursts repeated once every 40 ms (25 Hz) for 2 min per stimulation, as described previously (16). The participant or hospital staff member applied a conductive gel to the stimulation surfaces and applied nVNS to the region of the neck skin where the vagus nerve is located (i.e., between the trachea and the sternocleidomastoid muscle, above the palpable carotid pulse). Stimulation intensity (voltage) was increased until participants experienced noticeable platysma muscle contractions, as described previously (16). For prophylactic treatment, participants or hospital personnel administered 2 consecutive 2-min doses of nVNS (1 on each side of the neck) 3 times daily (morning, midday, and evening). Similar dosing schemes have been used to target peripheral pathophysiology in patients with postoperative ileus, gastroparesis, and Sjogren syndrome (17–19).

The SoC group was treated according to routine clinical standards for COVID-19 as defined by hospital protocol. Hospital protocol evolved rapidly as the pandemic progressed. Early on, patients remained on mechanical ventilators with no specific additional treatments. As the pandemic progressed, treatment protocols evolved to incorporate glucocorticoids, antivirals, monoclonal antibodies, and prone mechanical ventilation. This led to a range of SoC treatments being used in this study. Both groups were enrolled concurrently as per the randomization scheme, and there were no differences in SoC treatments between the groups (data not shown).

### Assessments and Outcomes

Efficacy and safety were evaluated *via* the incidence of specific clinical events and levels of cytokines and other biomarkers of inflammation, as summarized in Table 2. Blood-borne biomarkers were assessed in the clinical laboratory at the trial site using standard of care tests. Feasibility was evaluated *via* the number of stimulation sessions per participant per day, in which 1 session of stimulation comprised of two 2-min stimulations, 1 on each side of the neck. Because this was the first study of nVNS use in a new disease state, all outcomes were considered exploratory. Participants were assessed for adverse events (AEs) throughout the study, and the relationship between the AEs and the use of nVNS was assessed.

**Table 2:** Summary of study assessments.

**Assessed every study day**	**Assessed on days 1, 3, 5, and/or final study day^**a**^**
No. of nVNS doses administered	PaO_2_/FiO_2_
Dyspnea (yes/no)	Cytokines (TNF-α, IL-6, IL-1β)
Oxygen saturation	Procalcitonin
ICU admission (yes/no)	CRP
Need for ventilation (yes/no)	D-dimer
Survival (yes/no)	CBC (absolute lymphocytes, white blood cell count)
Discharged (yes/no)	Coagulation
Blood pressure	SAA
Adverse events	Ferritin
	Haptoglobin

a *The final study day was the day of discharge from the hospital, referral to the ICU, start of mechanical ventilation, or study withdrawal for each participant*.

*CBC, complete blood count; CRP, C-reactive protein; FiO_2_, fraction of inspired oxygen; IL, interleukin; ICU, intensive care unit; nVNS, non-invasive vagus nerve stimulation; PaO_2_, partial pressure of oxygen; SAA, serum amyloid A; SaO_2_, oxygen saturation; TNF, tumor necrosis factor*.

### Statistical Analyses

As this was the first study of nVNS for the treatment of patients hospitalized with COVID-19, there are no previously published data on the anticipated effect size in humans, and a formal power analysis could not be performed.

The study protocol defined the safety population as all participants who signed the consent form and were enrolled in the study and the intention-to-treat population as all participants who were randomly assigned and, if in the nVNS group, used nVNS for at least 1 session. However, not all enrolled patients had data collected; thus, the evaluable population (i.e., enrolled participants who signed the informed consent form and had baseline/demographic and medical history data collected) was used for analysis. Within the evaluable population, data for all endpoints at all time points were not obtained from every participant owing to the inherent challenges associated with executing a study in the rapidly changing treatment environment of the pandemic. Thus, the number of participants with observed laboratory data varied by analyte and is denoted for each. Imputation for missing data was not performed. Results of complete case analyses are presented. In addition, given the paucity of observed data after day 5, analyses are limited to data collection from baseline through day 5 of follow-up.

Participant demographics, baseline characteristics, and medical history were summarized for the sample as a whole and by treatment group using descriptive statistics. Treatment groups were compared using chi-square or Fisher exact tests (as appropriate) for categorical variables and *t* tests for continuous variables. Change from baseline in in fraction of inspired oxygen (FiO_2_), oxygen saturation, systolic and diastolic blood pressure (BP), and all laboratory assessments were analyzed using a repeated-measures approach with treatment, day, and treatment-by-day interaction as fixed categorical effects and baseline value and age as fixed continuous covariates. An unstructured (co)variance structure was used to model within-patient correlation. For FiO_2_, oxygen saturation, systolic BP, and diastolic BP, day 0 was used as baseline, and day 1 was used as baseline for all laboratory assessments. The least squares mean (LSM) at day 5 and overall (ie, across all postbaseline data points collected through day 5, combined) was calculated for each treatment group. Differences between treatment groups were compared using *F* tests. The percentages of patients with C-reactive protein (CRP) levels <10 mg/L at days 1, 3, and 5 in each group were compared using chi-square test or Fisher exact tests, as appropriate. Length of hospital and intensive care unit stays were compared between treatment groups using the Wilcoxon rank sum test. Statistical analyses were performed with SAS 9.4 (SAS Institute Inc.). Values of *p* < 0.05 were considered statistically significant. There were no adjustments for multiple comparisons.

## Results

### Participants

**Figure 3:**
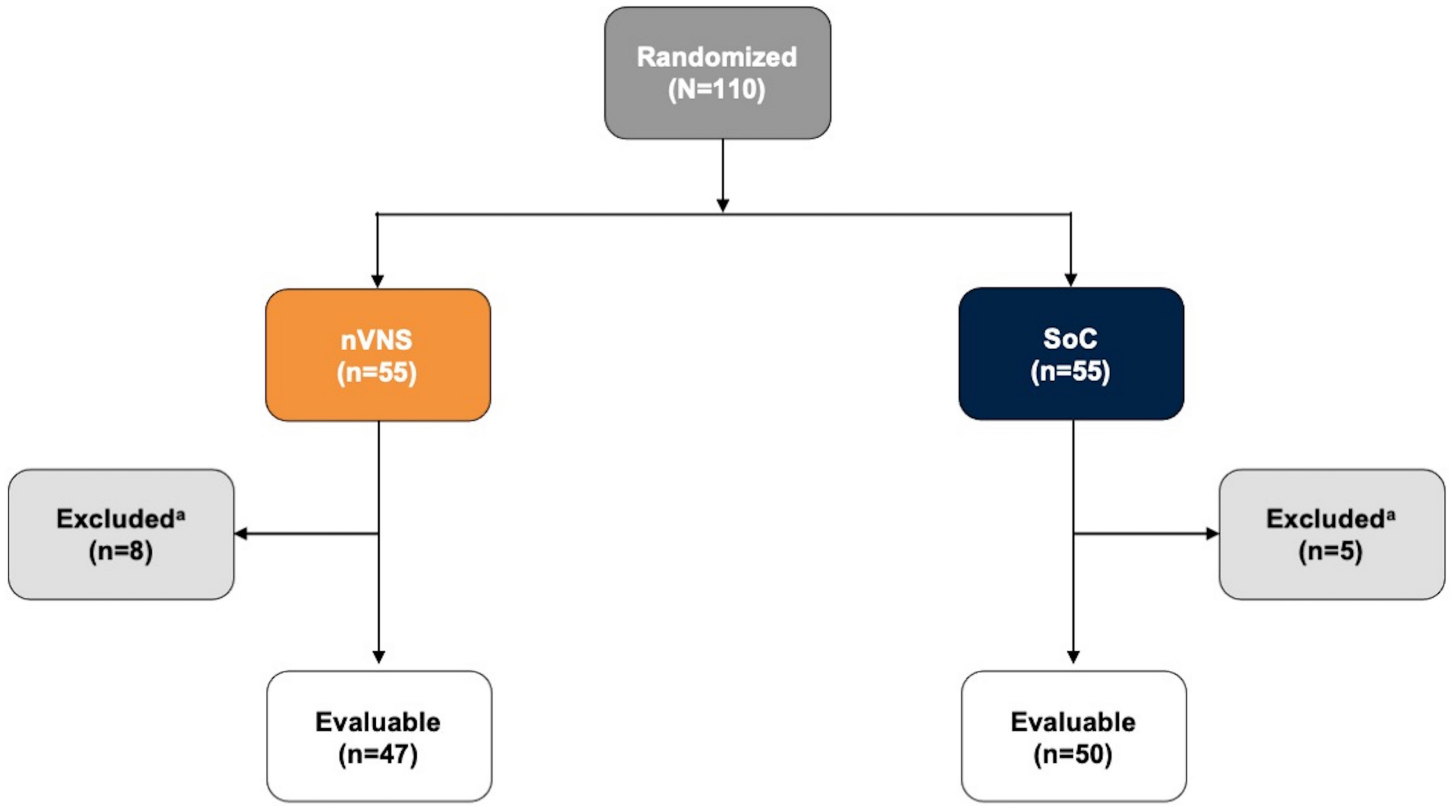
Participant Disposition. ^a^Excluded participants had missing baseline demographic and/or medical history data. nVNS, non-invasive vagus nerve stimulation; SoC, standard of care.

The study began on March 31, 2020, and was completed on February 23, 2021. One hundred ten participants were enrolled and randomly assigned, 55 to each group; among these, 97 (47 in the nVNS group and 50 in the SoC group) had baseline/demographic and medical history data available and represented the evaluable population; those without baseline/demographic and medical history data available were excluded from analysis (Figure 3). The mean participant age was higher in the SoC group than in the nVNS group, but baseline characteristics were otherwise similar between the groups (Table 3). Both arms of the study were recruited and consented concurrently as per the randomization scheme, and no statistically significant differences in SoC were noted between groups (Table 3). Seven participants in the nVNS group were severely ill, whereas only 2 participants in the SoC group were severely ill. All but 2 participants, both in the nVNS group, were also diagnosed with pneumonia.

**Table 3:** Participant demographics, baseline characteristics, and relevant medical history.

	**nVNS** **(*n =* 47)**	**SoC** **(*n =* 50)**	**Total** **(*n =* 97)**
**Age**, mean (SD),^a^ y	55.5 (13.9)	61.3 (10.3)	58.5 (12.5)
**Gender, no. (%)**			
Female	15 (31.9)	12 (24.0)	27 (27.8)
Male	32 (68.1)	38 (76.0)	70 (72.2)
**Smoker, no. (%)**	1 (2.1)	1 (2.0)	2 (2.1)
**Drinking** **>2 cups/d, no. (%)**	0 (0.0)	2 (4.0)	2 (2.1)
**Severity of COVID-19, no. (%)**			
Mild^b, e^	24 (51.1)	28 (56.0)	52 (53.6)
Moderate^c, e^	16 (34.0)	20 (40.0)	36 (37.1)
Severe^d, e^	7 (14.9)	2 (4.0)	9 (9.3)
**Comorbidity, no. (%)**			
Arterial hypertension	16 (34.0)	25 (50.0)	41 (42.3)
Ischemic heart disease	2 (4.3)	3 (6.0)	5 (5.2)
Diabetes I/II	10 (21.3)	11 (22.0)	21 (21.6)
Dyslipidemia	22 (46.8)	19 (38.0)	41 (42.3)
Renal insufficiency	3 (6.4)	2 (4.0)	5 (5.2)
**Medications, no. (%)**			
Antibiotics (last 3 months)	7 (14.9)	7 (14.0)	14 (14.4)
Antihypertensives	17 (36.2)	23 (46.0)	40 (41.2)
Aspirin	6 (12.8)	9 (18.0)	15 (15.5)
Corticosteroids	7 (14.9)	7 (14.0)	14 (14.4)
Insulin	5 (10.6)	4 (8.0)	9 (9.3)
Inhaler	11 (23.4)	7 (14.0)	18 (18.6)
Oral hypoglycemic drugs	10 (21.3)	11 (22.0)	21 (21.7)
Statins	14 (29.8)	18 (36.0)	32 (33.0)

*COVID-19, coronavirus disease 2019; FiO_2_, inhaled oxygen fraction; nVNS, non-invasive vagus nerve stimulation; PaO_2_, partial blood pressure of oxygen; SoC, standard of care*.

a *Age differed significantly between groups (p = 0.022)*.

b *Defined by a PaO_2_ between 200 and 300 and an FiO_2_ ≤ 3 needed to maintain 92% oxygen saturation*.

c *Defined by a PaO_2_ between 100 and 200 and an FiO_2_ between 0.3 and 0.5 needed to maintain 92% oxygen saturation*.

d *Defined by a PaO_2_ ≤ 100 and an FiO_2_ >0.5 needed to maintain 92% oxygen saturation*.

e *Classifications were based on Hospital Clínico Universitario de Valencia adjudication of Spanish Ministry of Health guidance at the time of study initiation*.

### Feasibility

Over the first 5 days of hospitalization, 82% to 94% of subjects received all 3 stimulation sessions per day (median, 2.8; SD, 0.8; Supplementary Table 1).

### Efficacy

**Figure 4:**
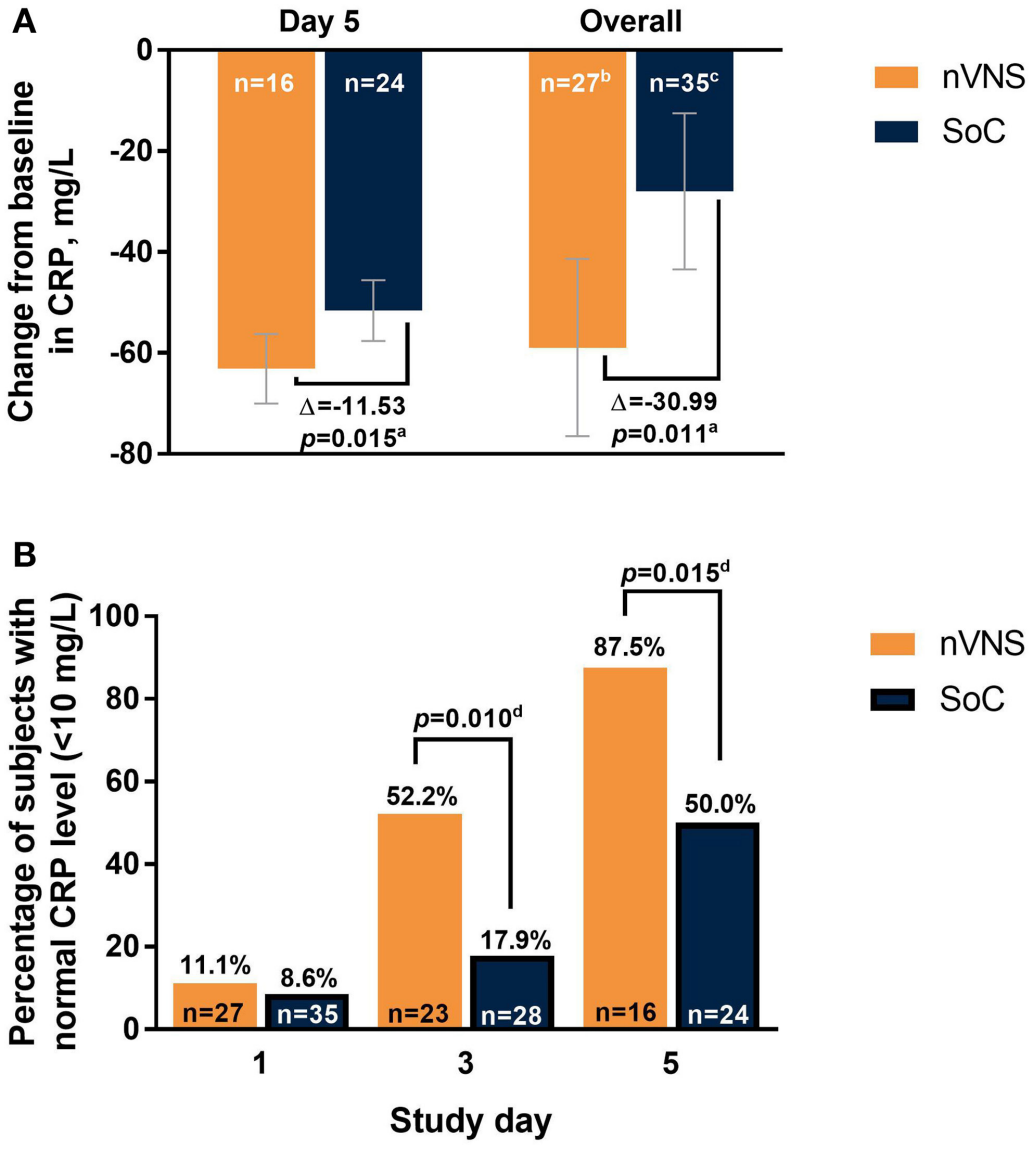
Changes From Baseline in CRP Level **(A)** and Percentage of Participants With Normal CRP Levels (<10 mg/L) **(B)** by Treatment Group Among Hospitalized Patients With COVID-19. Error bars denote 95% CIs. ^a^*p* values are from *F* tests comparing LSM changes from baseline for the nVNS and SoC groups. ^b^39 observations from 27 participants. ^c^52 observations from 35 participants. ^d^*p* values are from chi-squared or Fisher exact test, as appropriate. COVID-19, coronavirus disease 2019; CRP, C-reactive protein; LSM, least squares mean; nVNS, non-invasive vagus nerve stimulation; SoC, standard of care.

**Figure 5:**
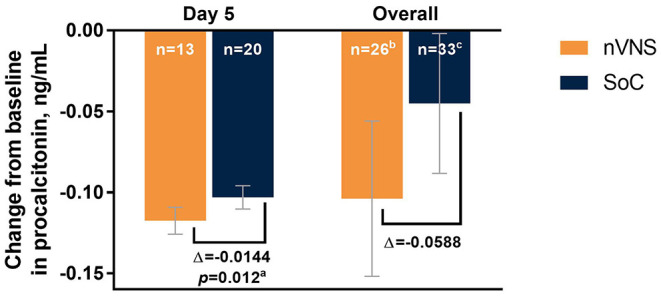
Changes From Baseline in Levels of Procalcitonin by Treatment Group Among Hospitalized Patients With COVID-19. Error bars denote 95% CIs. ^a^*p* values are from *F* tests comparing LSM changes from baseline for the nVNS and SoC groups. ^b^34 observations from 26 participants. ^c^45 observations from 33 participants. COVID-19, coronavirus disease 2019; LSM, least squares mean; nVNS, non-invasive vagus nerve stimulation; SoC, standard of care.

**Figure 6:**
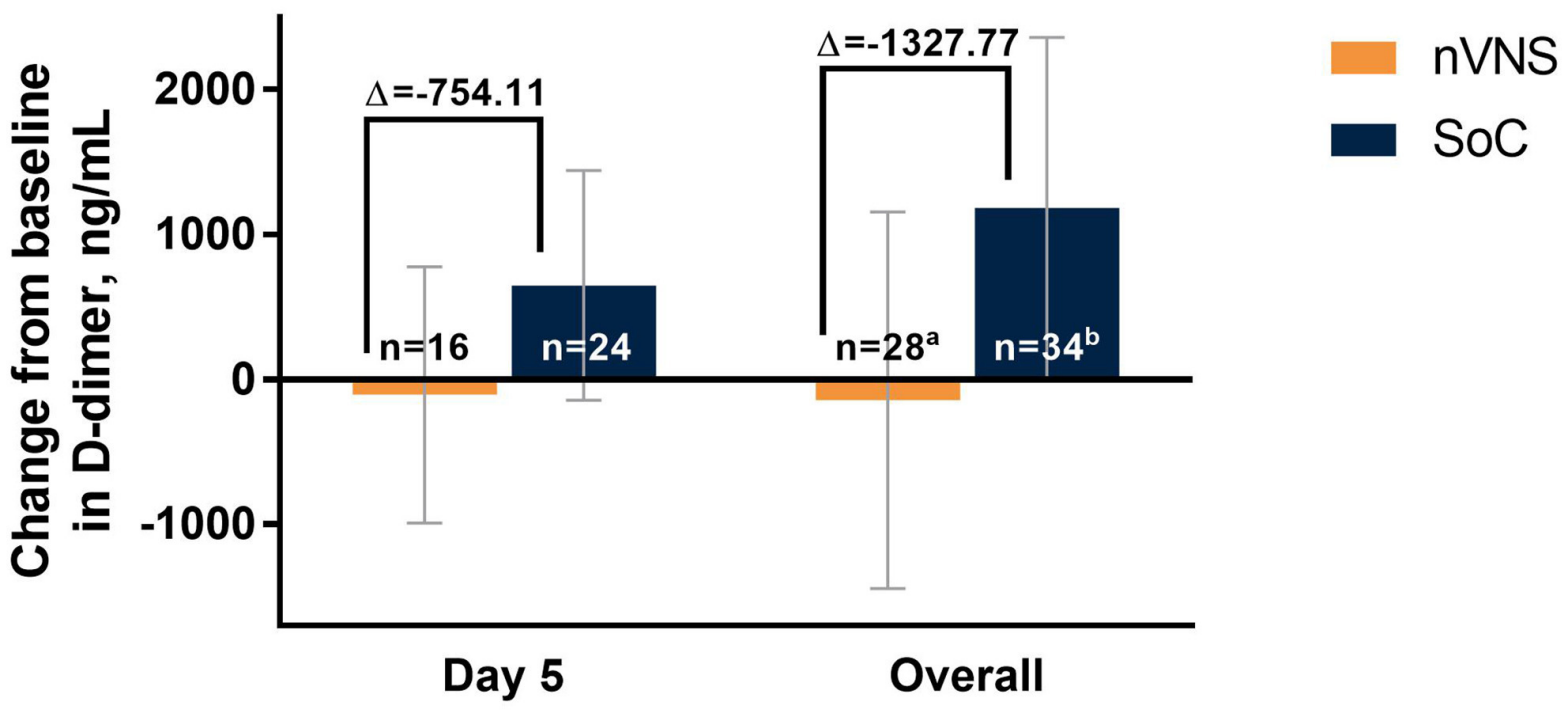
Changes From Baseline in Levels of D-Dimer by Treatment Group Among Hospitalized Patients With COVID-19. Error bars denote 95% CIs. ^a^40 observations from 28 participants. ^b^50 observations from 34 participants. COVID-19 indicates coronavirus disease 2019; LSM, least squares mean; nVNS, non-invasive vagus nerve stimulation; SoC, standard of care.

The analyses identified significant treatment differences for certain biomarkers of inflammation. Decreases from baseline in CRP levels were significantly greater for the nVNS group than for the SoC group at day 5 and overall (i.e., all postbaseline data points collected through day 5, combined; Figure 4A). At baseline (day 1), CRP levels were normal (<10 mg/L) for very few participants in both treatment groups, but by days 3 and 5 the percentage of participants with normal CRP levels had increased markedly and was significantly greater in the nVNS group than in the SoC group (Figure 4B). Compared with the SoC group, the nVNS group had a significantly greater decrease from baseline in procalcitonin level at day 5 (Figure 5). At day 5 and overall, levels of D-dimer were decreased from baseline for the nVNS group and increased from baseline for the SoC group, although the difference between the treatment groups did not reach statistical significance (Figure 6). Spaghetti plots illustrating each participant's change over time for CRP, procalcitonin, and D-dimer are presented in Supplementary Figures 1–3. No significant treatment differences were seen for the other clinical or biochemical markers that were evaluated (Supplementary Table 2).

### Safety

Overall changes in BP were assessed using 163 postbaseline observations from 46 participants in the nVNS group and 224 observations from 50 participants in the SoC group. Diastolic BP showed little change from baseline in the nVNS group (LSM, −0.22 mm Hg) and increased slightly in the SoC group (LSM, 1.70 mm Hg), with the difference between groups being statistically significant (−3.06 mm Hg; *p* = 0.02; data not shown). Changes from baseline in systolic BP were small in both the nVNS group (LSM, −0.93 mm Hg) and the SoC group (LSM, 0.31 mm Hg), with no significant difference noted between groups (−1.24 mm Hg; *p* = 0.611).

No serious nVNS-related AEs occurred during the study. In the context of the COVID-19 treatment ward, data on individual non-serious AEs were not recorded.

## Discussion

Preclinical studies first initiated nearly 20 years ago demonstrated that VNS is capable of modulating inflammation *via* the cholinergic anti-inflammatory pathway in experimental bacterially induced sepsis (9, 10, 20–22). However, the invasive nature of the surgical intervention required to apply direct VNS did not support the translation of this treatment to the clinical setting of sepsis or cytokine storm. In 2017, FDA approval of the first cervical non-invasive vagus nerve stimulator made it possible to investigate whether nVNS could reduce systemic inflammation in critical illness. Coincidentally, the development of nVNS technology was originally predicated on vagus nerve regulation of bronchoconstriction, the evidence for which led to the FDA to issue an EUA for nVNS use in adults with known or suspected COVID-19 (23, 24).

Several research groups have suggested transcutaneous cervical or auricular VNS as a potential approach for treating respiratory symptoms and modulating the cytokine storm associated with COVID 19 (23, 25–33). In patients with COVID-19, elevated inflammatory biomarker levels predict poor prognoses and clinical outcomes, including respiratory failure and mortality (34–36). High CRP levels (>40 mg/mL) are predictive of severe disease and mortality, whereas lower CRP levels are associated with less risk of disease progression (35). Procalcitonin and D-dimer also have been suggested as biomarkers of poor outcomes in COVID-19 (37). Procalcitonin levels have been found to be >4 times greater in patients with severe illness and up to 8 times greater in those with critical illness than in patients with moderate illness (38), and elevated procalcitonin values have been associated with a longer duration of mechanical ventilation (39). The current study was the first to systematically evaluate the effects of non-invasive cervical VNS on inflammation associated with a replicating virus in humans. Although the study was exploratory in nature, its results suggest significant modulation of CRP by nVNS as early as day 3, with a more robust effect by day 5. There was also evidence for modulation of procalcitonin levels by nVNS, with a significant effect observed on day 5. D-dimer levels also decreased (although not significantly) with nVNS.

Another clinical challenge associated with acute COVID-19 is a hypercoagulable state and an accompanying increased risk of stroke, heart attack, and chilblain-like lesions or “COVID toes” (40–42). Elevated levels of CRP, procalcitonin, and D-dimer each individually have been shown to be predictive of thrombotic complications, critical illness, and/or death during hospitalization for COVID-19 (43). Together, results from several recent studies suggest that elevations in CRP, procalcitonin, and/or D-dimer levels are associated with more severe disease and greater mortality (34–36, 38, 43–45). In the current study, differences between the nVNS and SoC groups in changes in D-dimer levels approached statistical significance, with an increase noted in the SoC arm and a slight decrease seen in the nVNS arm.

As the COVID-19 pandemic continues and new variants emerge, the need for additional treatment options persists. Our findings suggest that nVNS has mechanistic effects in acute COVID-19 that have potential applicability to address other aspects of the pandemic (e.g., early treatment to prevent the progression of symptoms or treatment for symptoms of post-acute sequelae of COVID-19). Our findings also suggest that nVNS is a feasible therapeutic modality for in-hospital use.

Studies of nVNS in asthma and chronic obstructive pulmonary disease have suggested that nVNS can be beneficial for the relief of acute bronchoconstriction and respiratory distress (23, 46, 47). Additionally, published case reports of 2 patients who used nVNS to manage respiratory symptoms of COVID-19 described rapid relief from chest congestion, chest tightness, and dyspnea as well as discontinuation of opioid and cough suppressant medications after nVNS therapy (23). These findings provided the impetus to conduct this prospective randomized study to investigate the effects of nVNS in patients with COVID-19 across a broad range of clinical and biochemical endpoints. Although no clear pulmonary benefits were noted, any potential acute benefit of nVNS on oxygen saturation likely was obscured by the inconsistency in the timing of such measurements relative to application of nVNS. Measurement of oxygen saturation or fraction of inspired oxygen (F_I_O_2_) immediately before and immediately after nVNS application would have been more prudent but would have placed an additional burden on health care resources that were already strained. Similarly, we were not able to control for supplemental oxygen, which would affect FiO_2_. Rapidly evolving treatment paradigms during the pandemic led to changes in the SoC during the course of the study, and no attempt was made to limit best clinical judgement. Both treatment groups received the SoC (with or without nVNS), which evolved concurrently in both groups, but no statistically significant differences in SoC were found between groups. Any strengths or weaknesses of the changing SoC should have been balanced between the groups and should not have led to non-comparable control group bias as recently described by Dodd et al. (48). This study did not control for experimental bias or placebo effects, which are recommended in a future study.

Under normal circumstances, all AEs that occur during a clinical trial are documented, and their relationship to the study treatment is carefully assessed. Our intention was to record and evaluate AEs in this manner, but this was not feasible under the circumstances of the COVID-19 pandemic. Although individual AEs were not tabulated, all investigators reported that, in their opinion, there were no serious AEs related to nVNS during the study. This is consistent with findings from controlled clinical trials in which >1000 participants were treated with nVNS and no serious treatment-related AEs were reported, establishing the exceptional safety and tolerability profile of nVNS (49).

As is common in early-stage investigations, we investigated a broad range of exploratory endpoints in this study. Consistent data collection was challenging in the environment of the pandemic, and the duration of hospitalization during the study varied widely (range, 3–42 days). As a result, many participants had missing data for several analytes at some sampling time points, and the limited number of long-term hospital stays precluded analysis beyond study day 5. In addition, there was some individual variability in values for CRP and procalcitonin. It should be noted that no nVNS dose response was assessed in this study, and no dose-finding studies have been performed in patients with known or suspected COVID-19. Despite these obstacles, nVNS therapy was associated with significantly greater improvements (vsSoC alone) in levels of the inflammatory biomarkers CRP and procalcitonin. The stimulation parameters used in this study (2 consecutive 2-min doses of nVNS [1 on each side of the neck] 3 times daily, for a total of 12 min of nVNS per day) were based on FDA clearances of gammaCore in migraine and cluster headache, which allow for up to 48 min of stimulation per day. The effects of nVNS therapy in patients with COVID-19 are being further investigated in another ongoing clinical trial (SAVIOR-II, ClinicalTrials.gov identifier: NCT04382391). A dose response of nVNS for the treatment of acute hemorrhagic and ischemic stroke is being investigated in a separate clinical trial (TR-VENUS, ClinicalTrials.gov identifier: NCT03733431).

## Conclusions

Novel treatment approaches are needed to address both acute COVID-19 and the emergent issue of post-acute COVID-19 syndrome. With multiple mechanisms of action that may be relevant to COVID-19, nVNS was granted an EUA to address its signs and symptoms. Our results show that nVNS therapy was feasible to implement in-hospital and was associated with significant reductions in levels of the inflammatory biomarkers CRP and procalcitonin. Although there was some suggestion that nVNS might affect D-dimer levels, wide confidence intervals rendered the results non-significant. This, along with the time required for appreciable degradation of D-dimer in a normal clinical setting after an infection, makes it difficult to draw conclusions on the role of nVNS in modulation of this biomarker. Nevertheless, as severe COVID-19 is characterized by inflammation and a hypercoagulable state, further study of these laboratory findings is warranted, as are investigations into the potential utility of nVNS early in the course of COVID-19 for possible prevention of significant symptoms and/or to address symptoms in patients with post-acute COVID-19 syndrome.

## Data Availability Statement

The data sets presented in this study can be found in online repositories. The names of the repository/repositories and accession number(s) can be found at: ClinicalTrials.gov with the identifier NCT04368156.

## Ethics Statement

The studies involving human participants were reviewed and approved by Ethics and Clinical Research Committee of INCLIVA, Valencia, Spain. INCLIVA is accredited as a Health Research Institute by the Carlos III Health institute and is affiliated with the Valencia University Clinical Hospital. The patients/participants provided their written informed consent to participate in this study.

## Author Contributions

CT, EP, MG, JO, MF, IB, DC, RV, PS, and EL contributed to conception and design of the study. CT, EP, MG, JO, MF, and IB acquired the data. CT, EP, MG, JO, MF, IB, DC, RV, CM, CC, EL, and PS participated in the analysis and interpretation of the data. All authors contributed to manuscript revision, read and approved the submitted version, and agreed to be accountable for all aspects of the work in ensuring that questions related to the accuracy or integrity of any part of the work are appropriately investigated and resolved.

## Funding

electroCore, Inc. (Rockaway, NJ, USA) developed the gammaCore Sapphire™ device and provided devices at no cost for use in this study. Statistical analysis for the study conducted by North American Science Associates Inc. (Minneapolis, MN, USA) and editorial support from MedLogix Communications, LLC (Itasca, IL, USA) were funded by electroCore, Inc.

## Conflict of Interest

DC has served as a consultant and advisory board member for Medtronic, Inc. RV is director of research for the National Spine and Pain Centers, a consultant and advisory board member for Medtronic, Inc., and chief executive officer for SGX Medical, LLC. CM is an employee of NAMSA, Inc., CC is a consultant for electroCore, Inc. and an employee of Convergent Medical Technologies, Inc., EL is an employee of electroCore, Inc., and receives stock ownership. PS is an employee and was cofounder of electroCore, Inc., and receives stock ownership. Author CC was employed by company Convergent Medical Technologies, Inc. The remaining authors declare that the research was conducted in the absence of any commercial or financial relationships that could be construed as a potential conflict of interest.

## Publisher's Note

All claims expressed in this article are solely those of the authors and do not necessarily represent those of their affiliated organizations, or those of the publisher, the editors and the reviewers. Any product that may be evaluated in this article, or claim that may be made by its manufacturer, is not guaranteed or endorsed by the publisher.
