# Supplementary material for: Non-invasive Vagus Nerve Stimulation for COVID-19: Results From a Randomized Controlled Trial (SAVIOR I)
Source: Front Neurol. 2022 Apr 8;13:820864. doi: 10.3389/fneur.2022.820864 (PMC9028764; doi:10.3389/fneur.2022.820864)
Supplement: Supplementary file 1 [file Data_Sheet_1.docx]

| **Supplemental Table 1.** Stimulation Use | | |
| --- | --- | --- |
| **Day** | **No. of stimulation sessions per day, mean (SD)** | **Participants with 3 stimulation sessions per day, %** |
| 1 | 2.93 (0.9) | 86 |
| 2 | 2.77 (0.7) | 89 |
| 3 | 2.68 (0.7) | 82 |
| 4 | 2.68 (0.9) | 86 |
| 5 | 2.83 (0.7) | 94 |

| **Supplemental Table 2.** Overall Change From Baseline for Additional Efficacy Assessments | | | | | | | | |
| --- | --- | --- | --- | --- | --- | --- | --- | --- |
| **Assessment** | **nVNS** | | | **SoC** | | | **Treatment difference** | ***p* value** |
|  | **n** | **Obs** | **LSM change from BL (95% CI)** | **n** | **Obs** | **LSM change from BL (95% CI)** | **LSM (95% CI)** |  |
| FiO_2_ | 47 | 168 | 0.04 (–0.01 to 0.09) | 50 | 224 | 0.03 (–0.02 to 0.07) | 0.01  (–0.05 to 0.08) | 0.650 |
| Oxygen saturation, % | 47 | 168 | 0.41 (0.06-0.75) | 50 | 224 | 0.28  (–0.02 to 0.57) | 0.13  (–0.32 to 0.59) | 0.568 |
| Lymphocytes, no./µL | 27 | 39 | 689.56 (459.09-920.03) | 35 | 52 | 472.19 (273.05- 671.33) | 217.37  (–93.30 to 528.05) | 0.166 |
| Platelets, no./µL | 27 | 39 | 83191.27 (53475.70-112906.80) | 35 | 52 | 66150.90 (40576.65-91725.16) | 17040.37  (–22152.80 to 56233.57) | 0.387 |
| TNF‑α, pg/mL | 23 | 29 | –1.66  (–6.36 to 3.04) | 28 | 39 | –2.26  (–6.06 to 1.55) | 0.60  (–5.48 to 6.67) | 0.849 |
| IL-6, pg/mL | 24 | 32 | 25.07  (–80.60 to 130.73) | 31 | 44 | 57.70  (–34.35 to 149.75) | –32.64  (–172.81 to 107.53) | 0.642 |
| Ferritin, ng/mL | 24 | 30 | –343.68  (–485.18 to  –202.17) | 30 | 41 | –214.93  (–339.39 to  –90.48) | –128.74  (–317.20 to 59.71) | 0.176 |
| Haptoglobin, mg/dL | 22 | 28 | –104.31  (–141.69 to  –66.92) | 29 | 40 | –66.63  (–99.05 to  –34.22) | –37.67  (–87.54 to 12.20) | 0.135 |
|  | **n** | **Mean (SD)** | | **n** | **Mean (SD)** | |  |  |
| Length of hospital stay, mean (SD), d | 47 | 11.0 (8.0) | | 49 | 10.1 (6.7) | | NA | 0.840^a^ |
| Length of ICU stay, mean (SD), d | 8 | 8.0 (6.1) | | 2 | 7.0 (1.4) | | NA | 0.933^a^ |
| *p* values are from *F* tests comparing the change from baseline overall (ie, for all postbaseline observations) for the nVNS and SoC groups unless otherwise indicated.  ^a^From Wilcoxon rank sum test.  Abbreviations: BL, baseline; FiO_2_, fraction of inspired oxygen; IL, interleukin; LSM, least squares mean; NA, not applicable; nVNS, non-invasive vagus nerve stimulation; obs, observations; SoC, standard of care; TNF, tumor necrosis factor. | | | | | | | | |
|  | | | | | | | | |

| **Supplemental Figure 1.** Individual CRP levels on study days 1, 3 and 5 among hospitalized patients with COVID-19 (A). Changes from baseline in levels of CRP among hospitalized patients with COVID-19 (B). |
| --- |
| A  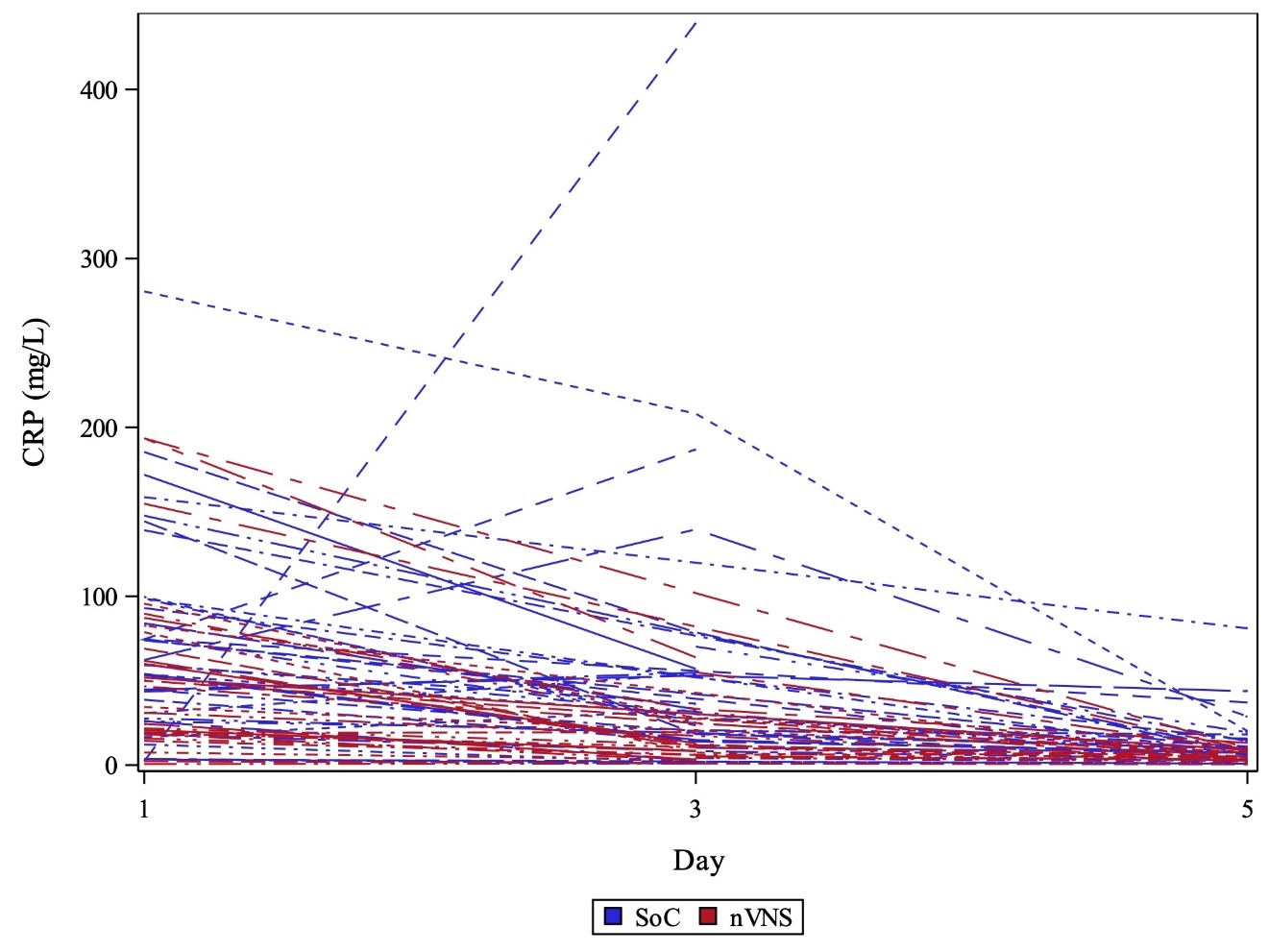 |
| B  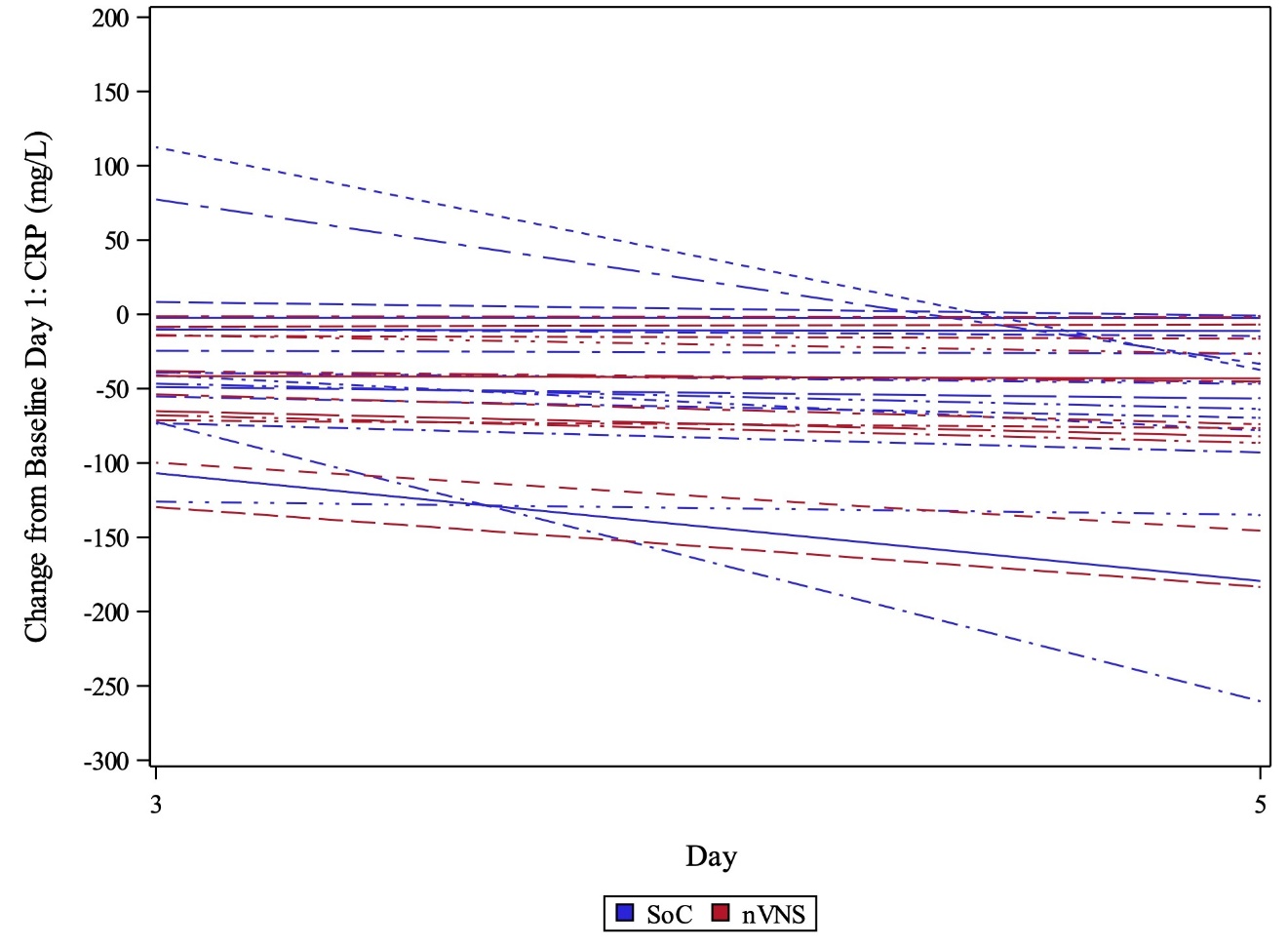 |
| Abbreviations: COVID-19, coronavirus disease 2019; CRP, c-reactive protein; nVNS, non-invasive vagus nerve stimulation; SoC, standard of care. |

| **Supplemental Figure 2.** Individual procalcitonin levels on study days 1, 3 and 5 among hospitalized patients with COVID-19 (A). Changes from baseline in levels of procalcitonin among hospitalized patients with COVID-19 (B). |
| --- |
| A  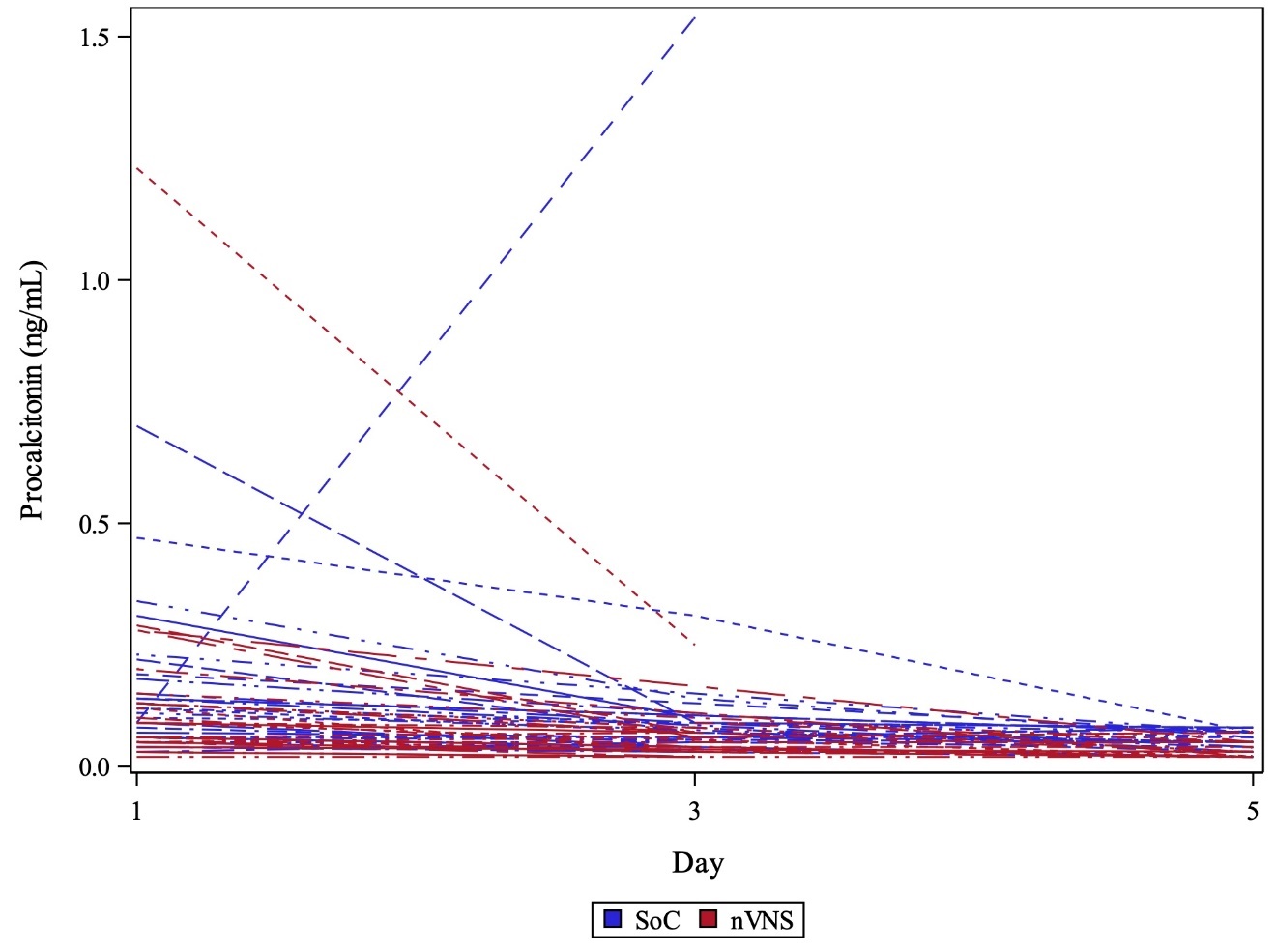 |
| B  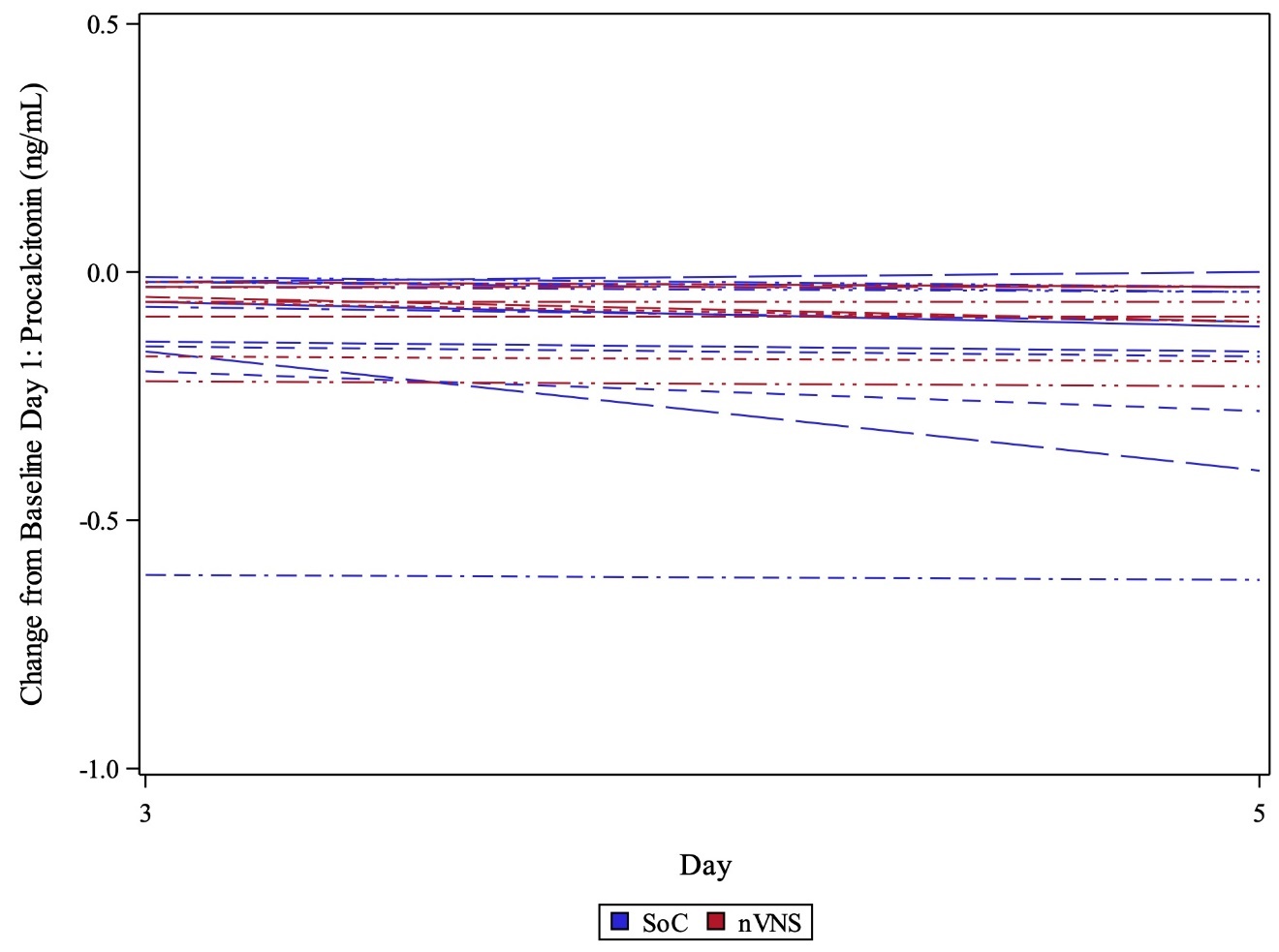 |
| Abbreviations: COVID-19, coronavirus disease 2019; nVNS, non-invasive vagus nerve stimulation; SoC, standard of care. |

| **Supplemental Figure 3.** Individual D-dimer levels on study days 1, 3 and 5 among hospitalized patients with COVID-19 (A). Changes from baseline in levels of D-dimer among hospitalized patients with COVID-19 (B). |
| --- |
| A  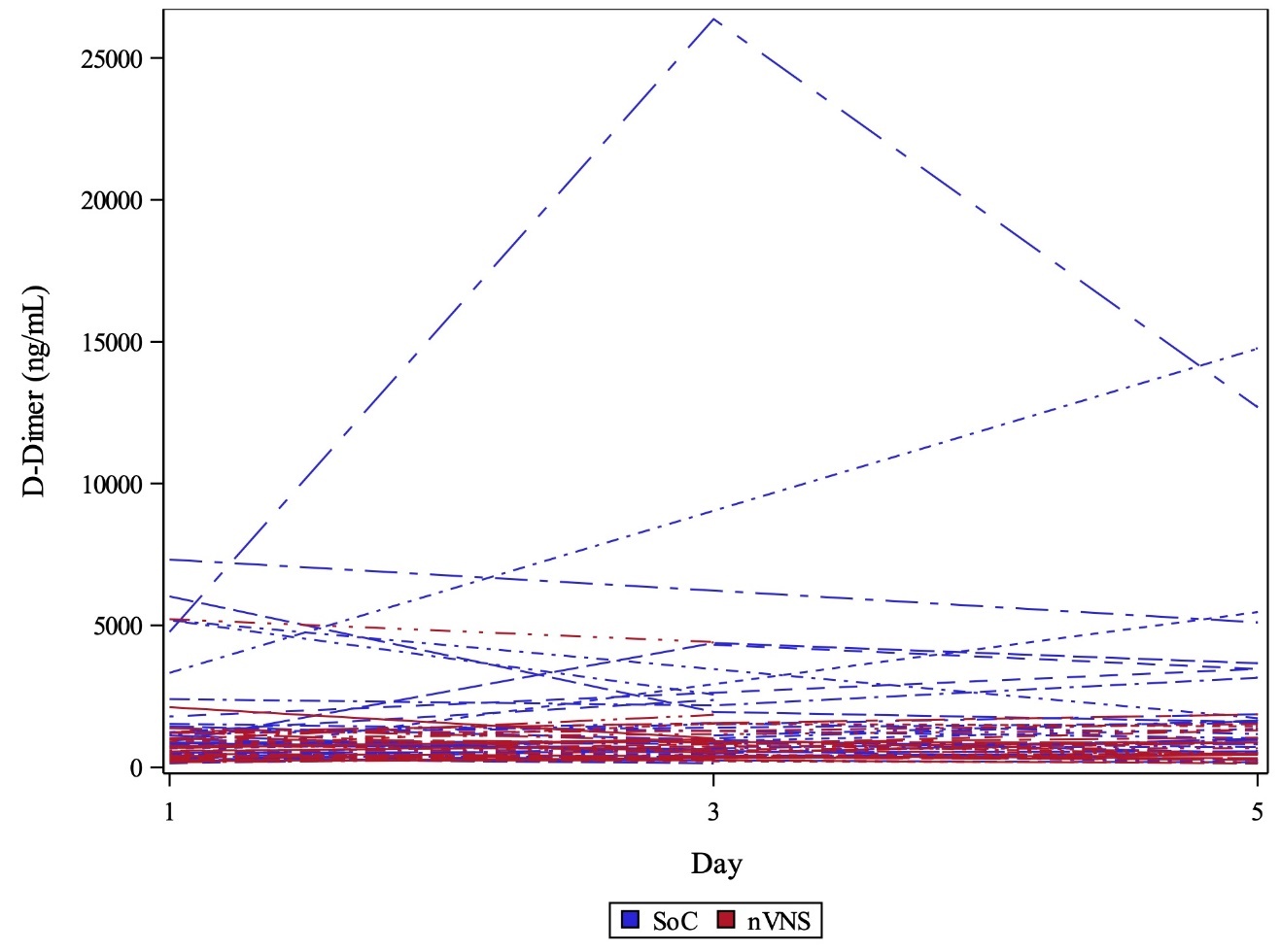 |
| B  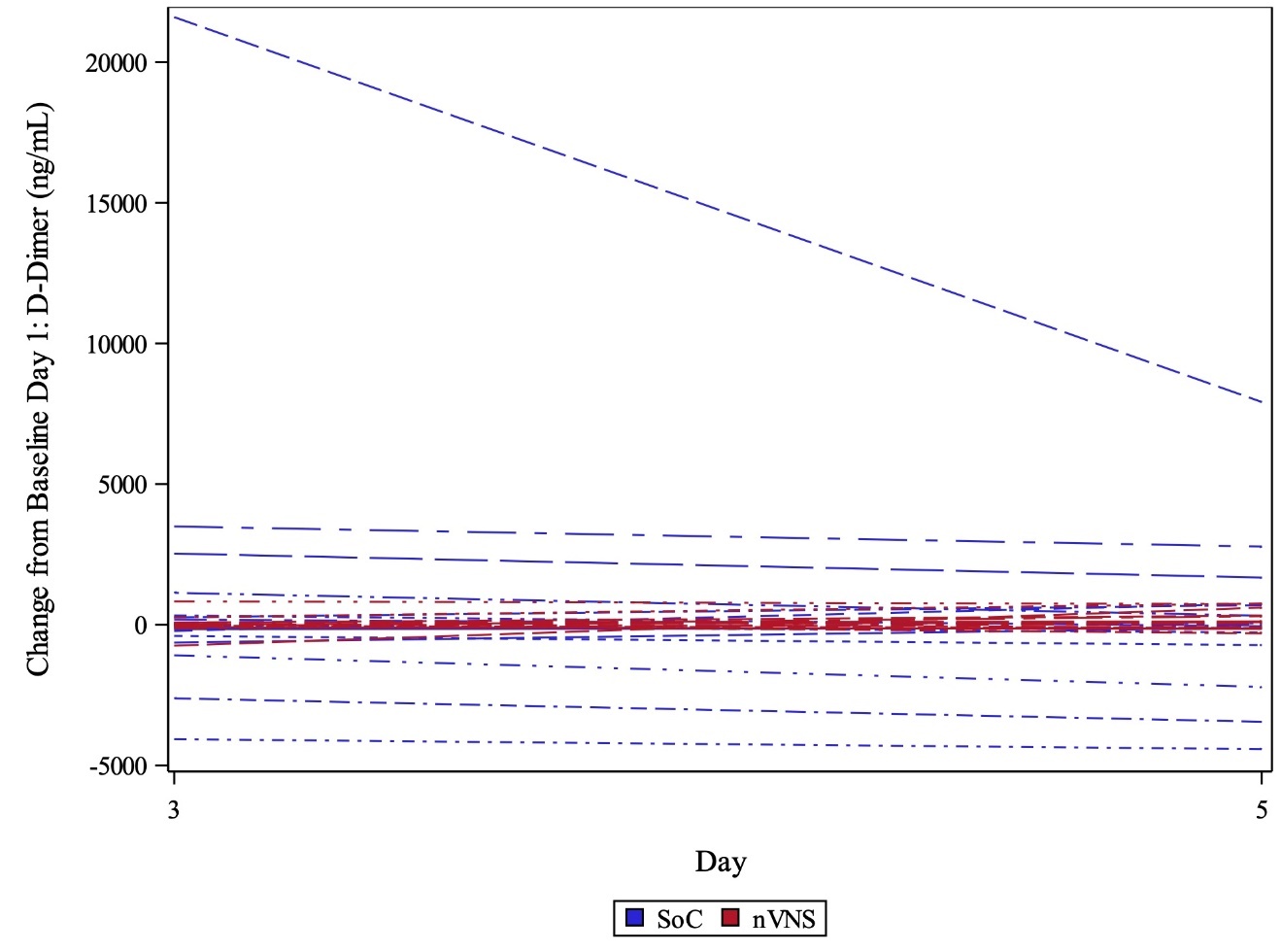 |
| Abbreviations: COVID-19, coronavirus disease 2019; nVNS, non-invasive vagus nerve stimulation; SoC, standard of care. |
